# Zearalenone and Metabolites in Livers of Turkey Poults and Broiler Chickens Fed with Diets Containing Fusariotoxins

**DOI:** 10.3390/toxins12080525

**Published:** 2020-08-15

**Authors:** Didier Tardieu, Angelique Travel, Jean-Paul Metayer, Celeste Le Bourhis, Philippe Guerre

**Affiliations:** 1 ENVT, Université de Toulouse, F-31076 Toulouse, France; didier.tardieu@envt.fr; 2 ITAVI, Centre INRAE Val de Loire, 37380 Nouzilly, France; travel@itavi.asso.fr; 3 ARVALIS-Institut du Végétal, Station expérimentale, 91720 Boigneville, France; M.Vilarino@Arvalisinstitutduvegetal.fr; 4 INRAE Unité Expérimentale 1295 PEAT, Centre INRAE Val de Loire, 37380 Nouzilly, France; celeste.lebourhis@inra.fr

**Keywords:** zearalenone, metabolites, tissues, UHPLC-MS/MS, turkeys, broilers

## Abstract

Zearalenone (ZEN) and metabolites were measured in livers of turkeys and broilers fed a control diet free of mycotoxins, a diet that contained 0.5 mg/kg ZEN (ZEN diet), and a diet that contained 0.5, 5, and 20 mg/kg of ZEN, fumonisins, and deoxynivalenol, respectively (ZENDONFB diet). The feed was individually distributed to male Grade Maker turkeys from the 55th to the 70th day of age and to male Ross chickens from the 1st to the 35th day of age, without any signs of toxicity. Together, the free and conjugated forms of ZEN, α- and β-zearalenols (ZOLs), zearalanone (ZAN), and α- and β-zearalanols (ZALs) were measured by UHPLC-MS/MS with [^13^C_18_]-ZEN as an internal standard and immunoaffinity clean-up of samples. ZAN and ZALs were not detected. ZEN and ZOLs were mainly found in their conjugated forms. α-ZOL was the most abundant and was found at a mean concentration of 2.23 and 1.56 ng/g in turkeys and chickens, respectively. Consuming the ZENDONFB diet significantly increased the level of total metabolites in the livers of chickens. Furthermore, this increase was more pronounced for the free forms of α-ZOL than for the conjugated forms. An investigation of the presence of ZEN and metabolites in muscle with the methods validated for the liver failed to reveal any traces of these contaminants in this tissue. These results suggest that concomitant dietary exposure to deoxynivalenol (DON) and fumonisins (FB) may alter the metabolism and persistence of ZEN and its metabolites in the liver.

## 1. Introduction

Zearalenone (ZEN) is a xeno-estrogenic mycotoxin produced by different fungi of the genus *Fusarium* [1,2]. Investigations of the worldwide contamination of cereals revealed maximum concentrations of ZEN in maize and wheat that often exceeded 1000 µg/kg, with 10–70% of samples contaminated, depending on the cereal and its geographic origin [1,3]. Although the toxic effects of ZEN are numerous, reproductive disorders appear to be the critical end point in humans and animals [1,2]. Accordingly, a tolerable daily intake (TDI) of 0.25 µg/kg body weight (b.w.) per day was adopted by the European Food Safety Agency to characterize consumer risk, and recommended levels were established for cereals used in animal feed [1,4].

After its administration in avian species, ZEN is rapidly absorbed [5,6]. Metabolism is intense, and many reduced and conjugated metabolites that vary considerably in their estrogenic potency are formed. Toxicity can be compared using the relative potency factors (RPFs) established in the uterotrophic assay, with ZEN having an RPF of one [1]. Alpha-zearalenol (α-ZOL) and beta-zearalenol (β-ZOL) are obtained by reduction of the C6′-ketonic carbonyl group of ZEN (Figure S1). The RPF of α-ZOL is 60, while the RPF of β-ZOL is 0.2. The reduction of the C1′ = C2′ double bond produces zearalanone (ZAN), which has an RPF of 1.5. The reduction of ZAN in the C6′-position generates alpha-zearalanol (α-ZAL) and beta-zearalanol (β-ZAL), whose RPFs are 4 and 2, respectively. Together, ZEN, ZAN, α-ZOL, β-ZOL, α-ZAL, and β-ZAL can be conjugated into sulfates and glucuronides on the phenolic hydroxyl groups in C2 or C4.

Surprisingly, although a TDI of ZEN has been established for human exposure, few data are available on the presence of ZEN and metabolites in tissues [1,2,7]. Because the conjugated forms of ZEN, ZAN, α-ZOL, β-ZOL, α-ZAL, and β-ZAL are not available as standards, most of the methods used to quantify ZEN and metabolites measure the conjugated forms after a step of enzymatic hydrolysis [8,9,10]. Because of the complexity of the matrices, ZEN and metabolites in tissues are often measured after cleaning up the samples on immunoaffinity (IA) columns [11,12,13,14,15,16,17,18]. Together, ZEN, ZAN, α-ZOL, β-ZOL, α-ZAL, and β-ZAL can be measured by UV or fluorescence detection, but the most recent methods use MS/MS detection. The chromatographic separation of ZEN and metabolites is frequently performed on C18 columns with gradient elution based on acidified water-acetonitrile (ACN) or acidified water-methanol (MetOH) [11,19,20,21,22,23,24,25]. An isotope-labeled internal standard ([^13^C_18_]-ZEN) is used for accurate quantitation [19,23,25].

The purpose of this study was to investigate the presence of ZEN and metabolites in avian species using different scenarios of exposure. Studies were performed in turkeys and broiler chickens to compare the metabolism of ZEN into α-ZOL and β-ZOL, which has been reported to vary across species [5,26]. Exposure was conducted at a low dose that did not demonstrate any signs of toxicity [27,28]. Because deoxynivalenol (DON) and fumonisins (FB) often co-occur in feed with ZEN, different diets were compared in each species, one containing ZEN alone and another containing ZEN, DON, and FB. This comparison was done to reveal whether a co-exposure to DON and FB, which are known for their effects on gut integrity, could change the amounts of ZEN and metabolites in tissues [29,30,31,32,33,34,35]. The liver was the main organ studied as it is known to be a target organ in terms of residues, while the detection of ZEN and metabolites was also carried out in muscles not reported to be contaminated with these toxins [7]. Tissue samples were spiked with [^13^C_18_]-ZEN, purified on an IA column, and analyzed by UPLC-MS/MS. As a result of the metabolism of ZEN in the avian species, accurate quantification of the free and conjugated forms of ZEN, α-ZOL, and β-ZOL and determination of the presence or absence of ZAN, α-ZAL, and β-ZAL were the key objectives of this analysis [6,7].

## 2. Results and Discussion

### 2.1. Separation of the Analytes and Matrix Interactions

**Figure 1:**
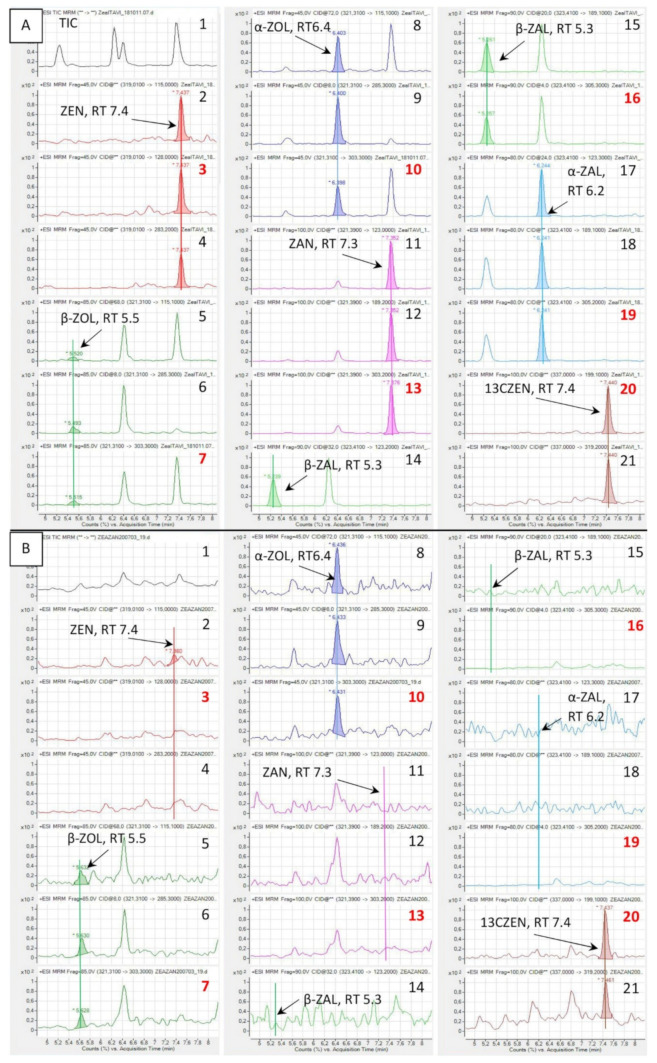
Typical chromatograms obtained using method 1. (**A**) Liver sample obtained from a turkey fed a diet free of ZEN, spiked after extraction with 25 ng/mL equivalent to 1 ng/g of each analyte. (**B**) Liver sample obtained from a turkey fed a diet containing ZEN at a concentration of 470 µg/kg for 14 days, spiked with 2.5 ng [^13^C_18_]-ZEN/g (13CZEN). 1: Total ion chromatogram (TIC); 2–21: ion masses, quantifiers are red typed; 2: 115; 3: ZEN 128; 4: 283.2; 5: 115.1; 6: 285.3; 7: 303.3; 8: 115.1; 9: 285.3; 10: 303.3; 11: 123; 12: 189.2; 13: 303.2; 14: 123.2; 15: 189.1; 16: 305.3; 17: 123.1; 18: 189.1; 19: 305.2; 20: 199.1; 21: 319.2.

Two methods were developed in this study. Method 1 enabled good separation of the seven analytes. Retention times ranged from 5.3 min for β-ZAL to 7.4 min for [^13^C_18_]-ZEN (Table S1). By contrast, the use of method 2 did not enable good separation of α-ZOL and ZAN, which have retention times of 6.5 and 6.6 min, respectively (Table S1). Because α-ZOL and ZAN have the same M+1 precursor weight and can lead to the same ions, the absence of separation of these two analytes could interfere with their detection [36]. Signal suppression and enhancement (SSE) for α-ZAL, α-ZOL, β-ZAL, β-ZOL, ZAN, and ZEN measured with method 1 ranged between 25% and 97% in liver and between 73% and 108% in muscle (Figure 1A, Table 1). By contrast, the SSE for α-ZOL, β-ZOL, and ZEN measured with method 2 ranged between 78% and 99% in liver and between 84% and 94% in muscle (Table 1). Strong matrix interactions have already been reported for ZEN and metabolites in tissues with QuEChERS pretreatment of samples and use of ACN in the mobile phase [21,22,37].

Signal-to-noise ratio (SNR) measured for α-ZAL, α-ZOL, β-ZAL, β-ZOL, ZAN, and ZEN with method 1 in blank liver extract spiked at 1.56 ng/mL was 6.5, 8.3, 4.7, 0.7, 4.5, and 2.4, respectively (Table 1). By contrast, the SNR measured for α-ZOL, β-ZOL, and ZEN with method 2 in blank liver extract spiked at 1.56 ng/mL was 7.3, 6.7, and 2.3, respectively (Table 1). A lower SNR for ACN when compared to MetOH is in agreement with data in the literature [37,38].

### 2.2. Recovery of the Analytes and Determination of the LOQ

The recovery of the analytes on blank spiked liver was measured for α-ZAL, α-ZOL, β-ZAL, β-ZOL, ZAN, and ZEN with method 1 at the concentration of 0.25 ng/g (Table 2). The extraction recovery (RE), apparent recovery, varied for α-ZAL, α-ZOL, β-ZAL, ZAN, and ZEN from 44% to 76% (Table 2). β-ZOL was not found in one spiked sample, and the RE of this analyte was only 16%. Correction of the concentration measured for α-ZAL, α-ZOL, ZAN, and ZEN by the rate of extraction measured for [^13^C_18_]-ZEN used as an internal standard in each sample allowed us to obtain satisfactory recovery levels for these analytes. A complementary factor was used for β-ZAL and β-ZOL to account for the SSE measured with these analytes (Table 1). With these corrections, a satisfactory recovery level was observed for β-ZAL but not for β-ZOL.

**Figure 2:**
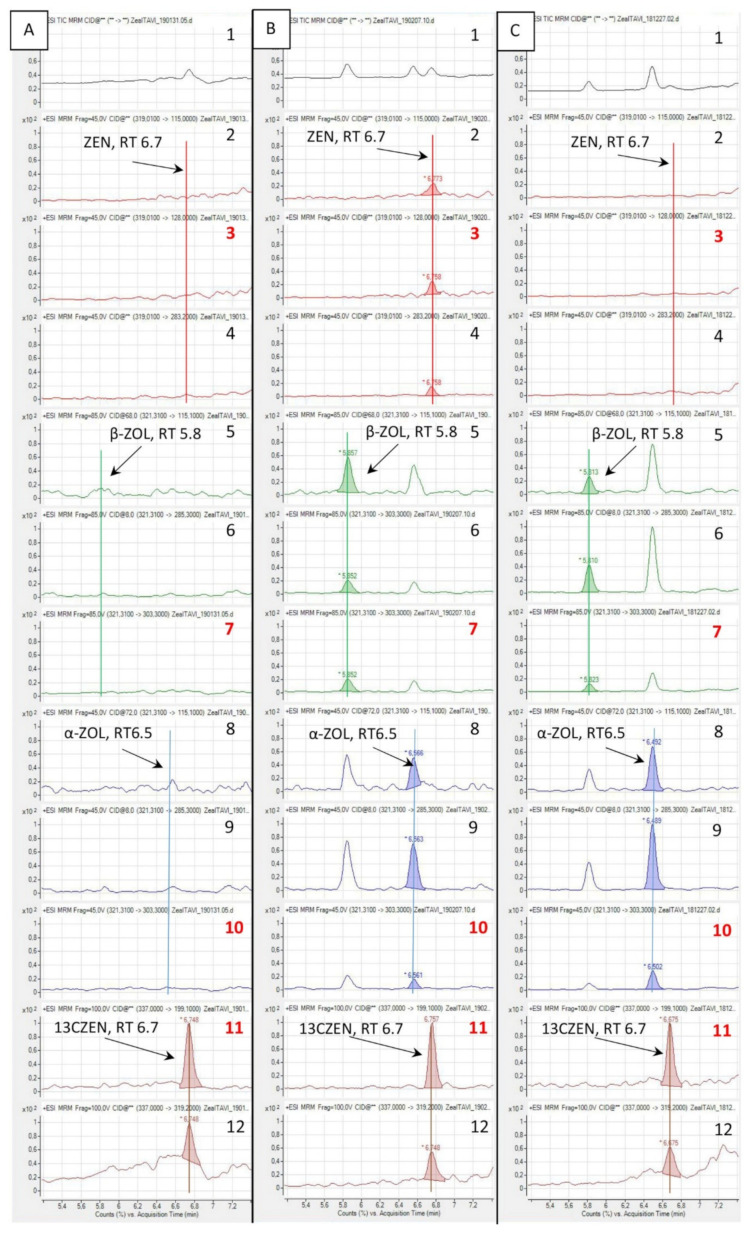
Typical chromatograms obtained from liver spiked with 2.5 ng [^13^C_18_]-ZEN/g (13CZEN) using method 2. Sample obtained from a turkey fed a diet free of ZEN (**A**) and spiked with 1 ng/g of each analyte (**B**). (**C**) Sample obtained from a turkey fed a diet containing ZEN at a concentration of 470 µg/kg for 14 days. 1: Total ion chromatogram (TIC); 2–12: ion masses, quantifiers are red typed; 2: 115; 3: 128; 4: 283.2; 5: 115.1; 6: 285.3; 7: 303.3; 8: 115.1; 9: 285.3; 10: 303.3; 11: 199.1; 12: 319.2.

The recovery of the analytes on spiked samples was measured for α-ZOL, β-ZOL, and ZEN with method 2. Figure 2A shows a typical UPLC-MS/MS chromatogram of a liver sample obtained from a turkey fed with a diet considered to be ZEN-free, spiked with 2.5 ng/g of [^13^C_18_]-ZEN and analyzed with method 2. No peak that could interfere with the detection of α-ZOL, β-ZOL, and ZEN was observed. Figure 2B is the same as Figure 2A, except liver was spiked before extraction with α-ZOL, β-ZOL, and ZEN, each at a concentration of 1 ng/g. As can be seen on this chromatogram, α-ZOL, β-ZOL, and ZEN are well separated and easy to quantify with acceptable ratios of the qualifiers. The extraction recovery (RE), apparent recovery, varied with the analyte from 52% to 62% (Table 3). Because the RE of β-ZOL was slightly higher that the RE of [^13^C_18_]-ZEN used as an internal standard, a correction factor of 0.88 was used for the measurement of the recovery in liver. The recovery of α-ZOL, β-ZOL, and ZEN in liver ranged from 92% to 116% (Table 3). Method 2 was linear over the range of concentrations assayed (Fisher test, *p* < 0.01 and *r*^2^ ≥ 0.99). The use of enzyme hydrolysis did not significantly change the recovery measured at 1 ng/g. The limit of quantitation (LOQ) for α-ZOL, β-ZOL, and ZEN was defined as the lowest validated level at 0.25 ng/g in liver using method 2. The relative standard deviation (RSD) of intra-day and inter-day assays ranged from 5% to 9%, and from 10% to 18%, respectively (Table 3).

Because of the strong SSE, the low SNR, and the low RE observed for β-ZOL in liver with method 1, and because a review of the literature revealed that α-ZAL, β-ZAL, and ZAN are not reported as being formed in avian species, it was decided that method 2 would be used to quantify α-ZOL, β-ZOL, and ZEN, while method 1 would be used to detect the presence or absence of α-ZAL, β-ZAL, and ZAN [6,7].

### 2.3. Detection of ZAN and ZALs

Method 1 was used to determine the presence or absence of α-ZAL, β-ZAL, and ZAN measured as the sum of the free and conjugated forms. Figure 1B shows a typical chromatogram of a liver sample obtained from a turkey fed a diet containing ZEN at a concentration of 470 µg/kg for 14 days, spiked with 2.5 ng/g of [^13^C_18_]-ZEN and analyzed using method 1. As shown in this figure, α-ZAL, β-ZAL, and ZAN were not detected, and these analytes were never found in any of the samples. These results are in agreement with a review of the literature, which revealed that ZAN and ZALs have never been characterized in avian species [6,7].

### 2.4. Total ZEN and ZOLs in Turkey Livers

Method 2 was used to quantify ZEN and ZOLs measured as the sum of the free and conjugated forms. Figure 2C shows a typical chromatogram of a liver sample obtained from a turkey fed a diet containing ZEN at a concentration of 470 µg/kg for 14 days, spiked with 2.5 ng/g of [^13^C_18_]-ZEN and analyzed using method 2. Although ZEN was present in the diet, no peak attributable to this analyte was found whereas α-ZOL and β-ZOL were easy to quantify. The concentrations of the total forms of α-ZOL, β-ZOL, and ZEN measured in livers of turkeys fed different diets are shown in Table 4. In the turkeys fed with the control diet that contained ZEN at a concentration of 35 µg/kg, only two liver samples contained α-ZOL, whereas no sample was positive for β-ZOL or ZEN. The concentrations of α-ZOL measured in the two positive samples were very low: below 0.5 ng/g. Some samples contained a peak that could be attributed to the quantifier used for ZEN, but none of these samples also contained acceptable values of the qualifiers. By contrast, in the livers of turkeys fed the diet containing ZEN at a level of 470 µg/kg, α-ZOL was quantifiable in all the samples and the mean concentration was 2.23 ng/g. Concerning β-ZOL, seven samples out of eight were quantifiable and the mean concentration was 1.14 ng/g. ZEN was quantified in only two samples, in one sample at a relatively high concentration.

The concentrations of α-ZOL, β-ZOL, and ZEN measured in turkeys fed with a diet containing ZEN at a concentration of 570 µg/kg plus DON and FB at the maximum recommended levels in poultry feed are reported in Table 4. Mean concentrations in α-ZOL, β-ZOL, and ZEN were 1.99, 1, and 0.5 ng/g, respectively. Comparison of the concentrations of α-ZOL and β-ZOL measured in turkeys fed the ZEN diet and in turkeys fed the ZENDONFB diet revealed no significant difference (ANOVA). In particular, the α:β ratio remained nearly constant in the two trials, at 1.96 and 1.99 with the ZEN and ZENDONFB diets, respectively. No statistical comparison was done for ZEN due to the small number of samples that were quantifiable.

Because the concentration of ZEN was not exactly the same in the two diets, a liver:feed ratio was calculated to account for this difference. This ratio was obtained by dividing the cumulated concentration of analytes in liver by the concentration of ZEN in feed. The difference in the molecular weight of ZEN and ZOL was considered negligible. The ratio was 8.3 × 10^−3^ in turkeys fed the ZEN diet and 6.1 × 10^−3^ in turkeys fed the ZENDONFB diet, respectively. The ratios did not differ, suggesting that the concomitant administration of DON and FB with ZEN has only weak consequences for final concentrations of ZEN and its metabolites in the livers of turkeys.

### 2.5. Total ZEN and ZOLs in Chicken Livers

Chromatograms obtained using method 2 for the analysis of chicken liver samples were similar to the one shown in Figure 2C. Table 5 lists the concentrations of total α-ZOL, β-ZOL, and ZEN measured in livers of chickens fed different diets. In chickens fed the control diet, only one liver sample was positive for α-ZOL but at a very low concentration. In the livers of chickens fed the diet containing ZEN alone, the mean concentration of α-ZOL was 1.56 ng/g, and all the samples were quantifiable. The mean concentration of β-ZOL was 0.8 ng/g, six samples out of eight were quantifiable, and one was positive but at a value below the LOQ. Concerning ZEN, only two samples were quantified with very low concentrations. The concentrations of α-ZOL, β-ZOL, and ZEN measured in the livers of chickens fed with a diet containing ZEN plus DON and FB were 2.57, 1.37, and 0.35, respectively. A statistical comparison of the concentrations of α-ZOL and β-ZOL measured in the livers of chickens fed the ZEN or the ZENDONFB diet revealed a significant difference between the two groups for α-ZOL (ANOVA, *p* = 0.029), but the difference was not significant for β-ZOL (Table 4). Interestingly, the α:β ratio did not differ in chickens fed the ZEN or the ZENDONFB diet, with respective values of 1.95 and 1.88. No statistical comparison was done for ZEN due to the small number of samples that were quantifiable.

The liver:feed ratio was calculated as described for turkeys. This ratio was 5.7 × 10^−3^ in chickens fed the ZEN diet and 10.3 × 10^−3^ in those fed the ZENDONFB diet. These ratios differed strongly (ANOVA, *p* = 0.01), suggesting that the concomitant administration of DON and FB with ZEN increased total concentrations of ZEN and its metabolites in the livers of chickens.

### 2.6. Hydrolysis of the Conjugates and Comparison of the Free Forms

**Figure 3:**
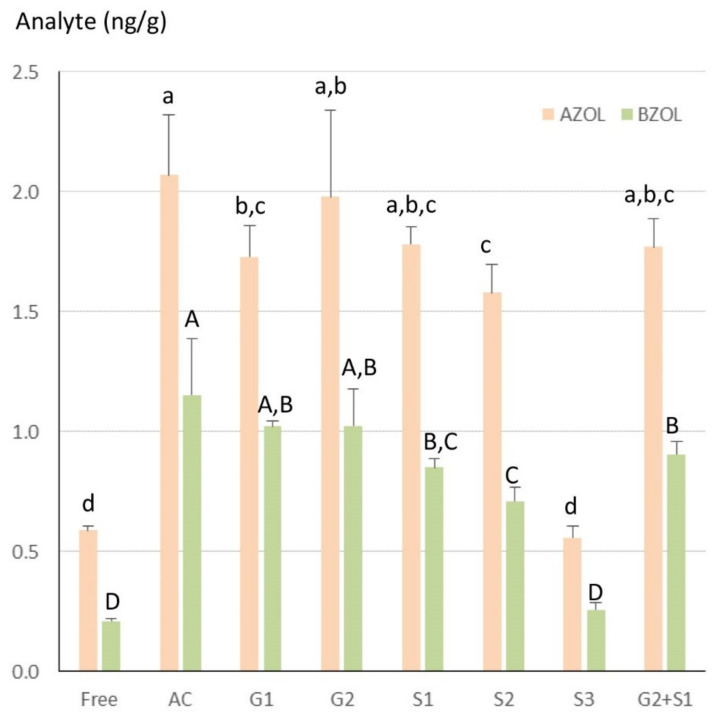
Amount of free and total α-ZOL and β-ZOL measured in a homogenate of livers obtained from turkeys fed a diet containing ZEN at a concentration of 470 µg/kg for 14 days. Results are expressed as mean ± SD of 5 determinations. Free = control, without hydrolysis of the conjugated forms, AC = acid hydrolysis, G1 = type H-1 β-glucuronidase from *Helix pomatia,* G2 = type H-2 β-glucuronidase from *Helix pomatia*, S1 = type H-1 sulfatase from *H. pomatia*, S2 = type H-2 sulfatase from *H. pomatia*, S3 = type VI sulfatase from *Aerobacter aerogenes*. A between-group statistical difference was found (ANOVA). Different letters (a,A,b,B,c,C) indicate statistical differences between groups.

As shown in Figure 3, α-ZOL and β-ZOL were highly conjugated in the liver and the amount of ZOLs dosed differed according to the enzyme used for the hydrolysis of the conjugates. Together, acid hydrolysis (AC), H-1 and H-2 β-glucuronidase from *H. promatia* (G1 and G2, respectively), and H-1 and H-2 sulfatase from *H. pomatia* (S1 and S2, respectively) led to a threefold increase in the concentrations of α-ZOL and β-ZOL compared to the free forms. By contrast, VI sulfatase from *Aerobacter aerogenes* (S3) was unable to hydrolyze the conjugates. Surprisingly, the use of G2 and S1 in combination did not increase the amount of α-ZOL and β-ZOL formed compared to the use of G2 or S1 alone. This result disagrees with previous data obtained at a very high level of ZEN [6,7]. Differences between studies could be due to the low level of ZEN in feed in this study while very high concentrations of enzymes were used. Indeed, the specifications of the enzymes used reveal that β-glucuronidase derived from mollusks also has sulfatase activity while sulfatase has a high β-glucuronidase secondary activity [39,40]. Taken together, these results suggest that care should be taken in making between-study comparisons of the hydrolyzed forms [1,7,8,41,42,43]. Also, these results reveal that the conjugated forms of ZOLs are easy to hydrolyze under acid conditions. This result is interesting because the conjugated forms of ZEN are considered as detoxified metabolites in the animal that can be hydrolyzed in the human gut, contributing to ZEN toxicity [6,7,44,45,46,47].

**Figure 4:**
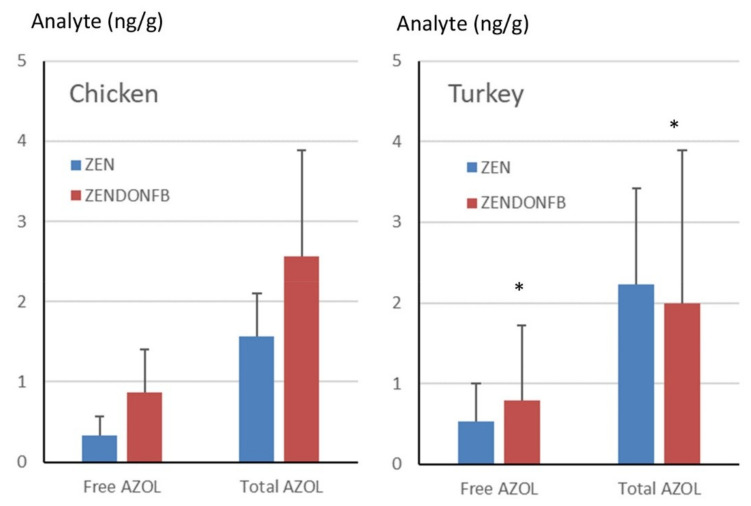
Amounts of free and total forms of α-ZOL measured in the livers of turkeys and chickens fed a diet containing ZEN alone or a diet of ZEN combined with DON and FB. Results are expressed as mean ± SD of 14 measurements. ANOVA was performed to identify the effect of the diet on the amount of free and total α-ZOL. Asterisks identify groups that differ statistically.

The amounts of free and total α-ZOL found in the livers of turkeys and chickens fed the contaminated diets are compared in Figure 4. In turkeys, free α-ZOL represented 24% of the total forms in animals fed the ZEN diet and 40% in animals fed the ZENDONFB diet, but this difference was not statistically significant. In chickens, the free forms of α-ZOL represented 21% of the total forms in animals fed the ZEN diet and 33.7% in animals fed the ZENDONFB diet (Figure 4). This difference was significant (ANOVA, *p* = 0.039), suggesting that the increase in the concentration of α-ZOL observed in the livers of chickens fed DON and FB was more pronounced for the free forms than for the conjugated forms. Comparison cannot be done for β-ZOL as only three samples in turkeys and four samples in chickens showed concentrations of the free forms above the LOQ.

### 2.7. General Discussion and Between-Study Comparison

A TDI of ZEN has been established, and evaluation of human exposure to this toxin and its metabolites is a priority in health risk assessment [1,2,7,47]. Surprisingly, only a few studies have been done at realistic exposure levels to investigate the presence of ZEN and its metabolites in animal tissues. This study was done to compare different scenarios of exposure in two avian species to highlight between-protocol convergences and divergences. No effects on health and performance were observed whatever the diet fed [27,28]. Special attention was given to the measurement of α-ZOL and β-ZOL, which strongly vary in their toxicity [1,47]. The strong matrix interaction, low SNR, and low extraction recovery observed for β-ZOL with method 1 led to the use of two methods of analysis, one for the detection of positive samples and another for the quantitation of the free and total forms of ZEN and ZOLs in the positives.

Feeding ZEN alone or in combination with DON and FB in turkeys and chickens revealed that only traces of ZEN were detected in the liver, whereas relatively high levels of α-ZOL and β-ZOL were measured, mainly in their conjugated forms, and no traces of α-ZAL, β-ZAL, or ZAN were found. The high metabolism of ZEN and the lack of formation of α-ZAL, β-ZAL, and ZAN agree with what was found in other studies in avian species at different levels of exposure [5,6,7,8,26,42,48]. Concerning α-ZOL and β-ZOL, α-ZOL was always the most abundant in this study, in agreement with in vitro studies conducted in six avian species and studies conducted at a higher level of ZEN in turkeys [8,26]. Other studies conducted at a similar level of ZEN exposure to that used in this study revealed that α-ZOL was an important metabolite in poultry, but these studies failed to detect β-ZOL, probably because of the method of analysis used [41,42]. A significant correlation (Pearson, *p* < 0.05) was found between the concentrations of the total forms of α-ZOL and β-ZOL measured in livers of turkeys fed the ZEN and ZENDONNFB diets, with *r*^2^ values of 0.766 and 0.584, respectively. Similarly, α-ZOL and β-ZOL were correlated in chickens fed the ZEN and ZENDONFB diets, with *r*^2^ values of 0.457 and 0.633, respectively. Due to the low number of livers with ZEN values above the LOQ, no correlation was sought for this analyte. An α:β ratio of around 2 was observed whatever the scenario of exposure used. The slope of the linear regression measured between the concentrations of total α-ZOL and total β-ZOL in livers ranged from 0.3 to 0.63, confirming that α-ZOL was more abundant than β-ZOL in this study. This result disagrees with a toxicokinetic study conducted at a single oral dose of 3 mg ZEN/kg b.w. in chickens and turkeys, where α:β ratios of 0.15 in chickens and 0.75 in turkeys were reported in plasma [5]. This between-study difference could be due to the dose, the mode of administration of the toxin, and the duration of exposure. An investigation of the presence of ZEN and metabolites in muscle with the methods validated for the liver failed to reveal traces of these contaminants in this tissue. This result agrees with data in the literature [6,7].

The consequences of the feeding of ZEN combined with DON and FB on ZEN and its metabolites in liver were more pronounced in chickens than in turkeys. The liver:feed ratio was increased in chickens fed the ZENDONFB diet compared to chickens fed the ZEN diet. This interaction, which has never been studied to date, could be explained by the alteration in gut permeability that has been observed with DON and FB [29,30,31,32,33,34,35]. Interestingly, the feeding of DON and FB also increased the share of free α-ZOL in the total α-ZOL dosed, even though the difference was only significant in chickens. This effect can be explained by competition between DON and ZOLs for the enzymes involved in the formation of the conjugated forms [6,49,50,51]. The lack of consequences of DON and FB on the α:β ratio is not incoherent with this hypothesis. Indeed, at least two different enzymes are involved in the reductive metabolism of ZEN, none being known to be involved in the metabolism of DON and FB [6,7,26,52].

In conclusion, feeding ZEN to turkeys and chickens at dietary concentrations of around 0.5 mg/kg did not enable the detection of ZEN and its metabolites in muscle but relatively high levels were found in the liver. Only weak differences between species were observed in ZEN metabolism; α-ZOL was the most abundant metabolite found, followed by β-ZOL, both being mainly conjugated. Feeding DON and FB combined with ZEN changed the total amount of metabolites in liver and the form in which these metabolites were found in chickens. Further studies are necessary to determine which mechanisms are involved in these interactions.

## 3. Material and Methods

### 3.1. Analytes and Reagents

Standard solutions of ZEN, α-ZOL, β-ZOL, and [^13^C_18_]-ZEN with certified concentrations of each analyte were purchased from Biopure (Tulln, Austria). ZAN, α-ZAL, and β-ZAL were purchased from Sigma (Saint Quentin Fallavier, France). Enzymes used for hydrolysis of the conjugate were purchased from Sigma and were of types H-1 β-glucuronidase (G1) from *Helix pomatia* (Cat# G0751), H-2 β-glucuronidase (G2) from *Helix pomatia* (Cat# G0875), H-1 sulfatase (S1) from *Helix pomatia* (Cat# S9626), H-2 sulfatase (S2) from *Helix pomatia* (Cat# S9751), and VI sulfatase (S3) from *Aerobacter aerogenes* (Cat# S1629). The immunoaffinity columns used were “easi-extract zearalenone” columns purchased from R-Biopharm Rhone LTD (Glasgow, Scotland). All other reagents were obtained from Sharlab S.L. (Sentmenat, Barcelona, Spain).

### 3.2. Tissue Samples

All experiments with animals were approved on May 18, 2017 by the French Ministry of Higher Education and Research and registered under number 02032.01. Tissue samples were obtained from turkeys and chickens reared in individual cages and fed ad libitum with different diets containing fusariotoxins as previously described [27,28]. Briefly, the diets containing mycotoxins were formulated on a corn/soybean basis by incorporation of ground cultured toxigenic *Fusarium* strains to reach a ZEN concentration of 0.5 mg/kg and concentrations of ZEN, DON, and FB1+FB2 of 0.5, 5, and 20 mg/kg, respectively. The control diets were considered to be free of mycotoxins. The final concentrations of ZEN, DON, and FB, and the concentrations of other mycotoxins that could contaminate the diets, were measured by HPLC-MSMS according to AFNOR NFV03-110 as previously described, and did not vary much from the targeted concentrations [27,28,53]. Each of the experimental diets was distributed to 14 turkeys from the 55th to the 70th day of age and to 14 broilers from the 1st to the 35th day of age. At the end of the experiment, feed was removed for a period of 8 h to take into account the slaughterhouse practice; the animals were stunned by electrocution and killed by exsanguination. The liver and breast muscles were collected and stored at −80 °C until analysis.

### 3.3. LC/MS/MS Conditions

The UPLC MS/MS system was composed of a 1260 binary pump with an autosampler coupled to an Agilent 6410 triple quad (Santa Clara, CA, USA). Analytes were separated using a Poroshell 120 column (3.0 × 50 mm, 2.7 µm). Chromatograms were analyzed using MassHunter quantitative analysis software from Agilent. Detection was conducted after positive electrospray ionization with the following source parameters: gas temperature: 300 °C; gas flow: 10 L/minute; nebulizer: 25 psi; capillary voltage: 4000 V. The optimized multiple reaction monitoring (MRM) conditions used for each analyte are listed in Table S1. The most abundant transition was chosen for MRM quantitation of ZEN, ZAN, α-ZOL, β-ZOL, α-ZAL, and β-ZAL, while two other transitions were used as qualifiers. The quantifier used for [^13^C_18_]-ZEN was the ion with a mass weight of 199.1, which gives the best signal-to-noise ratio (SNR), and only one transition was used for qualification.

Two methods were developed for the detection and quantitation of the analytes. For method 1, the mobile phase was composed of a mixture of acetonitrile (ACN), used as solvent A, and water, used as solvent B, each containing 0.1% formic acid (*v*/*v*). Solvents A and B were 30% and 70%, respectively, at the beginning of the run. A gradient of elution was introduced to reach 70% of A at 0.5 min then to return to 30% of A at 13 min. A 5-min washing step with 30% of A was performed before each new run. For method 2, the mobile phase was composed of a mixture of methanol (MetOH), used as solvent A, and water, used as solvent B, each containing 0.1% formic acid (*v*/*v*). Solvents A and B were in the same proportion at the beginning of the run. A gradient of elution was introduced to reach 95% of A at 5 min then to return to 50% of A at 8 min. A 9-min washing step with 50% of A was performed before each new run. The mobile phase was delivered at a flowrate of 0.3 mL/min.

### 3.4. Analysis of Standard Solutions

Standards were diluted in acetonitrile and combined to obtain working solutions containing mixtures of ZEN, ZAN, α-ZOL, β-ZOL, α-ZAL, and β-ZAL for assays with method 1 and mixtures of ZEN, α-ZOL, and β-ZOL for assays with method 2. Variable volumes of working solutions were evaporated to dryness in conical polypropylene tubes. The dry residue was solubilized in 200 µL of a mixture of solvents A and B in the same proportions and sonicated for 1 min. The entire solution was transferred to an insert, which was placed in the autosampler. Ten microliters was injected. Expected concentrations of ZEN, ZAN, α-ZOL, β-ZOL, α-ZAL, and β-ZAL were 0, 1.56, 6.25, 25, and 100 ng/mL. A quadratic fit of the measured signal (*y*-axis) vs. concentration (*x*-axis) was used for each analyte at 0, 1.56, 6.25, 25, and 100 ng/mL. The method was linear (Fisher test, *p* < 0.01 and *r*^2^ ≥ 0.99) for a concentration range of 6.25 to 100 ng/mL for each analyte while quadratic adjustment allowed us to obtain acceptable accuracy at 1.56 ng/mL.

### 3.5. Matrix Interactions

Matrix interactions were measured for method 1 and method 2 on tissue samples obtained from animals not exposed to ZEN in their diet over a period of at least 14 days. Muscle and liver were prepared using the same protocol. Five grams of tissue was placed in a conical 50 mL polypropylene tube and homogenized in 5 mL of sodium acetate 0.2 M (pH 7.2) with an Ultra Turrax (IKA^®^-Werke GmbH & CO, Staufen, Germany). Twenty milliliters of acetonitrile and ten milliliters of hexane were added. The tubes were placed on a stir table for 15 min then in an ultrasonic bath for 15 min. The upper phase was discarded after 10 min of centrifugation at 2500× *g*. The lower phase was centrifuged for 10 min at 4000× *g*, and 20 mL of the supernatant fraction was collected and evaporated to dryness at 45 °C under a gentle stream of nitrogen to obtain dry residue N°1. Dry residue N°1 was suspended in 2.5 mL methanol, vortexed, and placed in an ultrasonic bath for 5 min. Then, 22.5 mL of phosphate buffered saline was added, and the mixture was vortexed and placed in an ultrasonic bath for 5 min. Solubilized extracts were passed through the IA column as described above to obtain dry residue N°2. Variable volumes of working solutions containing the analytes were added to dry residue N°2 and evaporated to dryness to obtain dry residue N°3. Dry residue N°3 was solubilized in 200 µL of mobile phase and injected. The matrix interactions for α-ZAL, α-ZOL, β-ZOL, β-ZAL, ZAN, ZEN, and [^13^C_18_]-ZEN were measured using method 1, while method 2 was used to measure the matrix interaction for α-ZOL, β-ZOL, ZEN, and [^13^C_18_]-ZEN. Expected concentrations of each analyte passed through the two methods were 0, 1.56, 6.25, 25, and 100 ng/mL. SSE was calculated using the following equation: (slope of the matrix-matched calibration curve/slope of the solvent-based calibration curve) × 100.

### 3.6. Recovery Rates

Method 2 was used to measure the recovery rates of solutions containing α-ZOL, β-ZOL, and ZEN that were spiked to liver samples obtained from animals not exposed to ZEN in their diet over a period of at least 14 days. Variable volumes of working solutions containing the analytes were placed in a conical 50 mL polypropylene tube and evaporated to dryness. Then, 5 g of tissue was added and treated to obtain dry residue N°1 as described for the measurement of matrix interaction. Dry residue N°1 was passed through the IA column to obtain dry residue N°2. Dry residue N°2 was solubilized in 200 µL of mobile phase and injected using the analytical conditions described for method 2. Expected concentrations of α-ZOL, β-ZOL, and ZEN were 0, 0.25, 1, and 5 ng/g each whereas the expected concentration of [^13^C_18_]-ZEN was 2.5 ng/g. The intra-day repeatability (*n* = 5) and inter-day reproducibility (5 days) were calculated for [^13^C_18_]-ZEN and expressed by the RSD of the concentrations measured. Lack of interaction due to the use of enzymes for the hydrolysis of the conjugates was checked at the concentration of 1 ng/g.

### 3.7. Metabolites in Tissues

#### 3.7.1. Total Metabolites

Total metabolites, which corresponded to the sum of the free and conjugated metabolites, were measured after hydrolysis of the conjugates. Five grams of tissue was placed in a conical 50 mL polypropylene tube, and the sample was treated as described above to obtain dry residue N°1. Dry residue N°1 was suspended in 5 mL acetate buffer (0.2M, pH 5), vortexed, and placed in an ultrasonic bath for 5 min. Enzyme hydrolysis was obtained by adding 100 µL of H-2 β-glucuronidase from *Helix pomatia* (1000 U), and the mixture was placed on a shaking bath at 37 °C. After incubation overnight, the mixture was placed in a conical 50 mL polypropylene tube that contained 12.5 µL of 1 µg/mL [^13^C_18_]-ZEN evaporated to dryness. Twenty milliliters of ACN was added, and the tube was placed on a stir table for 15 min then in an ultrasonic bath for 15 min. The tube was centrifuged at 4000× *g* for 10 min, and the supernatant fraction was collected and evaporated to dryness at 45 °C under a gentle stream of nitrogen to obtain dry residue N°1B. Dry residue N°1B was treated as described above for dry residue N°1 and passed through the IA column to obtain dry residue N°2. Dry residue N°2 was solubilized in 200 µL of mobile phase and injected in the chromatographic system using the analytical conditions described for method 1 and method 2. Total α-ZAL, β-ZAL, and ZAN were never detected while α-ZOL, β-ZOL, or ZEN was found in most of the liver samples but not in the muscle samples. The concentrations of α-ZOL, β-ZOL, and ZEN were calculated by quadratic adjustment using method 2.

#### 3.7.2. Free Metabolites

Free metabolites were measured in liver with method 2 as follows. For each sample, 12.5 µL of 1 µg/mL [^13^C_18_]-ZEN was placed in a conical 50 mL polypropylene tube and evaporated to dryness. Then, 5 g of liver was placed in the tube, and the sample was treated as described for the measurement of matrix interaction to obtain dry residue N°1. Dry residue N°1 was passed through the IA column to obtain dry residue N°2, which was solubilized in 200 µL of mobile phase and injected using the analytical conditions described for method 2. The concentrations of α-ZOL, β-ZOL, and ZEN were calculated by quadratic adjustment.

### 3.8. Comparison of Enzymes Used for the Hydrolysis and Acid Hydrolysis

Different enzymes and methods of hydrolysis of the conjugates were compared on a liver homogenate obtained from turkeys fed a diet containing ZEN at a concentration of 470 µg/kg for 14 days. Homogenate was treated in triplicate to obtain free α-ZOL, β-ZOL, and ZEN using the method described for the determination of free metabolites in tissues. Total α-ZOL, β-ZOL, and ZEN were obtained as described for the determination of total metabolites in tissues except that H-1 and H-2 β-glucuronidase (G2) from *Helix pomatia*, H-1 and H-2 sulfatase (S2) from *Helix pomatia*, and VI sulfatase (S3) from *Aerobacter aerogenes* were successively used for the step of enzymatic hydrolysis. Acid hydrolysis was conducted by suspending the dry residue N°1 in 5 mL HCl 0.1 M overnight, after which 100 µL NaOH 5M was added prior to ACN extraction and purification of the sample on an IA column as described for the determination of total metabolites in tissues. Extracts were analyzed using method 2, and the concentrations of α-ZOL, β-ZOL, and ZEN were calculated by quadratic adjustment.

### 3.9. Acceptability Parameters and Statistical Analysis

Accuracy was considered as acceptable for a relative standard deviation (RSD) of 20%. SSE of 80–120% was considered acceptable while values outside this range indicated a strong matrix effect. Variation of the ratio of the qualifier in samples had to be below 20% in comparison to the ratio of the qualifier measured in standards. Variation of the retention time in samples had to be below 5% in comparison to the retention time measured in standards.

The linearity of the calibration curves obtained with solutions of standards, calibration curves obtained after passage of the standard on an IA column, and calibration curves obtained to measure the matrix effect was statistically evaluated by the Fisher test (*p* < 0.01) and by the determination coefficient (*r*^2^), which had to be ≥ 0.99. The linearity of the method over the range of concentrations assayed was evaluated using the same methods. The statistical significance of the differences observed in the recovery rates, matrix effects, and metabolites in tissues was evaluated using one-way ANOVA after a normality test (Shapiro–Wilk) was done. When a significant difference was found (*p* < 0.05), a comparison of means was performed with the Duncan test. Different letters identify statistically different groups (*p* < 0.05). Correlations between total α-ZOL and total β-ZOL were performed by Pearson’s correlation coefficient test (*p* < 0.05). All statistical analysis was conducted by XLSTAT Biomed (Addinsoft, Bordeaux, France).

## Figures and Tables

**Table 1:** Signal suppression and enhancement and signal-to-noise ratio observed with the two methods in blank sample extracts.

	α-ZAL	α-ZOL	β-ZAL	β-ZOL	ZAN	ZEN	[^13^C_18_]-ZEN
Method 1							
SSE Liver (%) ^1^	88	87	63	25	84	75	97
SSE Muscle (%) ^1^	108	106	55	98	105	73	-
SNR Liver ^2^	6.5	8.3	4.7	0.7	4.5	2.4	-
SNR Muscle ^2^	7.4	3.2	3.2	2	3.1	3.1	-
Method 2							
SSE Liver (%) ^1^	-	83	-	99	-	80	78
SSE Muscle (%) ^1^	-	94	-	94	-	84	-
SNR Liver ^2^	-	7.3	-	6.7	-	2.3	-
SNR Muscle ^2^		1.9	-	2.1	-	1.7	-

^1^ Signal suppression and enhancement (SSE) measured in triplicate in blank sample extracts spiked after extraction at 6.25, 25, and 100 ng/mL equivalent to 0.25, 1, and 5 ng/g. ^2^ Signal-to-noise ratio (SNR) measured in blank sample extracts spiked after extraction at 1.56 ng/mL equivalent to 0.0624 ng/g.

**Table 2:** Recovery of the analytes after immunoaffinity clean-up of blank liver spiked at 0.25 ng/g using method 1.

	α-ZAL	α-ZOL	β-ZAL	β-ZOL	ZAN	ZEN	[^13^C_18_]-ZEN
Extraction Recovery (%) ^1^	76 ± 6	71 ± 8	44 ± 6	16 ± 11	70 ± 7	65 ± 10	57 ± 7
Recovery ^2^	0.30 ± 0.04 (120%)	0.28 ± 0.04 (113%)	0.27 ± 0.05 (107%)	0.32 ± 0.1 (127%)	0.28 ± 0.02 (111%)	0.26 ± 0.06 (104%)	

^1^ Mean ± SD measured in 5 blank liver samples (5 g) spiked with 0.25 ng/g of α-ZAL, α-ZOL, β-ZAL, β-ZOL, ZAN, and ZEN and with 2.5 ng [^13^C_18_]-ZEN/g. β-ZOL was not detected in 1 sample. ^2^ Concentrations of α-ZAL, α-ZOL, β-ZAL, β-ZOL, ZAN, and ZEN were calculated by taking the extraction recovery (RE) measured for [^13^C_18_]-ZEN on each sample into account and expressed in ng/g and in percent. Concerning β-ZAL and β-ZOL, a factor of correction that corresponded to the [^13^C_18_]-ZEN:β-ZAL SSE ratio and to the [^13^C_18_]-ZEN:β-ZOL SSE ratio was also used. No correction was made to account for the matrix effect for α-ZAL, α-ZOL, ZAN, and ZEN.

**Table 3:** Recovery of the analytes after immunoaffinity clean-up of blank liver spiked at different concentrations using method 2.

	α-ZOL	β-ZOL	ZEN	[^13^C_18_]-ZEN
Extraction Recovery (%) ^1^	55 ± 12	62 ± 10	52 ± 8	55 ± 9
Liver Spiked at 0.25 ng/g ^2^	0.29 ± 0.06 (116%)	0.28 ± 0.01 (110%)	0.25 ± 0.03 (101%)	
Liver Spiked at 1 ng/g ^2^	1.08 ± 0.05 (108%)	1.08 ± 0.09 (108%)	0.92 ± 0.09 (92%)	
Liver Spiked at 5 ng/g ^2^	5.20 ± 0.51 (104%)	5.11 ± 0.85 (102%)	4.60 ± 0.58 (97%)	
Liver Spiked at 1 ng/g with a Hydrolysis Step ^2,3^	0.96 ± 0.06 (96%)	0.98 ± 0.05 (98%)	0.84 ± 0.12 (84%)	
Intra-Day RSD (%)	5	7	9	
Inter-Day RSD (%)	10	14	18	

^1^ Mean ± SD measured in blank liver samples (5 g) spiked with 0.25, 1, and 5 ng/g of α-ZOL, β-ZOL, and ZEN and with 2.5 ng [^13^C_18_]-ZEN/g. ^2^ Concentrations of α-ZOL, β-ZOL, and ZEN were calculated by taking the RE measured for [^13^C_18_]-ZEN on each sample into account. Concerning β-ZOL, a factor of 0.88 that corresponded to the ratio of the extraction recovery rates measured for [^13^C_18_]-ZEN and β-ZOL was also used. No correction was made to account for the matrix effect. Data are expressed in ng/g and in percent. ^3^ Sample was treated with H-2 β-glucuronidase from *Helix pomatia* prior to extraction.

**Table 4:** Concentrations of α-zearalenol (α-ZOL), β-zearalenol (β-ZOL), and zearalenone (ZEN) in livers of turkeys fed with mycotoxin-contaminated diets. ^1^

	α-ZOL	β-ZOL	ZEN
Exposure (Days)	14	14	14
Feed Control (µg/kg) 55 to 70 d	<10	<10	35
Liver positive/total max; min (ng/g)	2/8 0.44; <0.25	0/8 <0.25	0/8 <0.25
Feed ZEN (µg/kg) 55 to 70 d	<10	<10	470
Liver (ng/g) mean ± SD max; min positive/total	2.23 ± 1.19 4.08; 0.55 8/8	1.14 ± 0.83 2.14; <0.25 8/8	0.54 ± 0.43 2.06; <0.25 4/8
Feed ZENDONFB (µg/kg) ^2^ 55 to 70 d	<10	<10	570
Liver (ng/g) mean ± SD max; min positive/total	1.99 ± 1.90 7.63; 0.63 8/8	1.00 ± 0.75 2.45; <0.25 8/8	0.50 ± 0.39 1.57; <0.25 5/8

^1^ Amounts of α-ZOL, β-ZOL, and ZEN are expressed as total metabolites analyzed with method 2. ^2^ Concentrations of DON, FB1, and FB2 were 5150, 21,500, and 4200 µg/kg, respectively.

**Table 5:** Concentrations of α-zearalenol (α-ZOL), β-zearalenol (β-ZOL), and zearalenone (ZEN) in livers of chickens fed with mycotoxin-contaminated diets. ^1^

	α-ZOL	β-ZOL	ZEN
Exposure (Days)	35	35	35
Feed Control (µg/kg) 1 to 10 d	<10	<10	35
11 to 35 d	<10	<10	25
Liver positive/total max; min (ng/g)	1/8 0.27; <0.25	0/8 <0.25	0/8 <0.25
Feed ZEN (µg/kg) 1 to 10 d	<10	<10	465
11 to 35 d	<10	<10	480
Liver (ng/g) mean ± SD max; min positive/total	1.56 ± 0.86 * 3.98; 0.52 8/8	0.80 ± 0.59 1.64; <0.25 7/8	0.27 ± 0.14 0.57; <0.25 3/8
Feed ZENDONFB (µg/kg) ^2^ 1 to 10 d	<10	<10	415
11 to 35 d	<10	<10	430
Liver (ng/g) mean ± SD max; min positive/total	2.57 ± 1.32 * 4.48; 0.76 8/8	1.37 ± 1.02 3.05; 0.52 8/8	0.35 ± 0.22 0.82; <0.25 3/8

^1^ Amounts of α-ZOL, β-ZOL, and ZEN are expressed as total metabolites analyzed with method 2. ^2^ Concentrations of DON, FB1, and FB2 were 3820, 17,600, and 1440 µg/kg in the diet fed from the 1st to the 10th day of age and 4170, 17,700, and 1530 µg/kg in the diet fed from the 11th to the 35th day of age, respectively. * Significant difference between the two groups (ANOVA, *p* = 0.029).

## Supplementary Materials

The following are available online at https://www.mdpi.com/2072-6651/12/8/525/s1, Figure S1: Relative estrogenic potency factors (RPFs) of zearalenone and its metabolites [1], Table S1: MRM transitions, MS/MS parameters, and retention times of the analytes.

## Abbreviations

ZEN	zearalenone
ZAN	zearalanone
ZOLs	zearalenols
α-ZOL	alpha-zearalenol
β-ZOL	beta-zearalenol
FB	fumonisins B
FB1	fumonisin B1
FB2	fumonisin B2
DON	deoxynivalenol
RPFs	relative potency factors
TDI	tolerable daily intake
b.w.	body weight
IA	immunoaffinity
G1	H-1 β-glucuronidase from *Helix pomatia*
G2	H-2 β-glucuronidase from *Helix pomatia*
S1	H-1 sulfatase from *Helix pomatia*
S2	H-2 sulfatase from *Helix* *pomatia*
S3	VI sulfatase from Aerobacter aerogenes
ACN	acetonitrile
MetOH	methanol
LC-MS/MS	liquid chromatography-tandem mass spectrometry
UPLC-MS/MS	ultraperformance liquid chromatography-tandem mass spectrometry
MRM	multiple reaction monitoring
RE	extraction recovery
SSE	signal suppression and enhancement
SNR	signal-to-noise ratio
RSD	relative standard deviation
LOQ	limit of quantitation
