# Supplementary material for: Zearalenone and Metabolites in Livers of Turkey Poults and Broiler Chickens Fed with Diets Containing Fusariotoxins
Source: Toxins (Basel). 2020 Aug 15;12(8):525. doi: 10.3390/toxins12080525 (PMC7472091; doi:10.3390/toxins12080525)
Supplement: Supplementary file 1 [file toxins-12-00525-s001.pdf]

Supplementary Materials: Zearalenone and Metabolites in Livers of Turkey Poults and Broiler Chickens Fed with Diets Containing Fusariotoxins

Didier Tardieu, Angelique Travel, Jean-Paul Metayer, Celeste Le Bourhis and Philippe Guerre

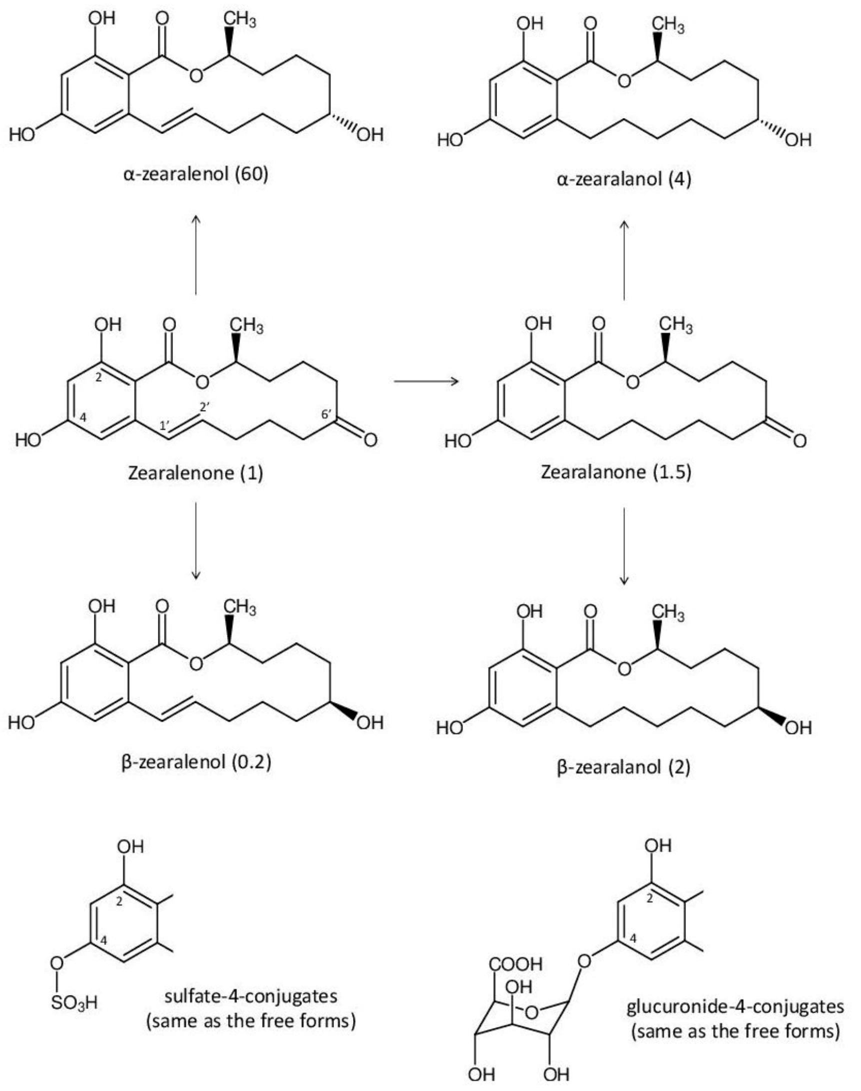

Figure S1. Relative estrogenic potency factors (RPF) of zearalenone and its metabolites [1].

Table S1. MRM transitions, MS/MS parameters and retention times of the analytes.

	**β-ZAL**	**β-ZOL**	**α-ZAL**	**α-ZOL**	**ZAN**	**ZEN**	**C13ZEN**
Precursor (M+1)	323.4	321.3	323.4	321.3	321.4	319	337
Quantifier	305.3	303.3	305.2	303.3	303.2	128	199.1
Fragmentation/Collision (V) [1]	90/4	85/0	80/4	45/0	100/8	45/64	100/16
Qualifier 1 (Abundance, %)	189.1 (34)	285.3 (56)	189.1 (26)	285.3 (69)	189.2 (20)	283.2 (90)	319.2 (125)
Fragmentation/Collision (V)	90/20	85/8	80/20	45/8	100/16	45/8	100/4
Qualifier 2(Abundance, %)	123.2 (18)	115.1 (16)	123.1 (13)	115.1 (21)	123 (13)	115 (99)	
Fragmentation/Collision (V)	90/32	85/68	80/24	45/72	100/32	45/68	
Retention time method 1 (min)	5.3	5.5	6.2	6.4	7.3	7.4	7.4
Retention time method 2 (min)	5.5	5.8	6.4	6.5	6.6	6.7	6.7

[1] Energies used for the fragmentation and collision.
